# Persistent negative symptoms in young people at clinical high risk of psychosis: findings from an Italian longitudinal study.

**DOI:** 10.1192/j.eurpsy.2025.410

**Published:** 2025-08-26

**Authors:** L. Pelizza, C. Ricci, E. Leuci, E. Quattrone, M. Menchetti, F. Catalano

**Affiliations:** 1 Department of Biomedical and Neuromotor Sciences, University of Bologna, Bologna; 2 Department of Mental Health, AUSL di Parma, Parma; 3 University of Bologna Italy, Bologna, Italy

## Abstract

**Introduction:**

Negative symptoms in CHR-P people are generally not responsive to treatments and commonly related to poorer functional outcome. However, less research attention has been dedicated to Persistent Negative Symptoms (PNS), defined as clinically stable negative symptoms of moderate severity evident for at least 6 months.

**Objectives:**

This study aims to (a) determine the prevalence of PNS in a sample of young people at CHR-P; (b) investigate any association of PNS with functioning and clinical features; (c) examine longitudinal course of PNS across 2 years of follow-up and changes in PNS severity levels with specialized treatments.

**Methods:**

One Hundred Eighty CHR-P participants were recruited and were divided into CHR-P/PNS + and CHR-P/PNS− subgroups according to the presence/absence of PNS. The clinical assessments were based on the PANSS and the GAF and were conducted at baseline and every 12 months during the follow-up. Association of PNS with sociodemopgraphic, clinical, psychopathological and treatment variables were examined using linear regression analysis.

**Results:**

Twenty four participants showed PNS at entry. Of them, 21 concluded the 2-year follow-up period. At baseline, the CHR-P/PNS + participants showed more educational and employment deficits, and more social and functioning impairment. During the follow-up, the CHR-P/PNS + subgroup had a significant longitudinal decrease in negative symptoms, which was specifically related to antidepressant treatment. CHR-P/PNS + subjects also showed a higher incidence of new hospitalization and a lower functional recovery over time.

**Conclusions:**

Our findings support that the persistence of negative symptoms in CHR-P people is longitudinally related to worse daily functioning and more severe clinical conditions that are at higher risk of hospitalization and are less responsive to specialized treatments.

**Disclosure of Interest:**

None Declared

